# Development and Characterization of Orally Disintegrating Tablets Containing a Captopril-Cyclodextrin Complex

**DOI:** 10.3390/pharmaceutics12080744

**Published:** 2020-08-07

**Authors:** Adina Magdalena Musuc, Valentina Anuta, Irina Atkinson, Vlad Tudor Popa, Iulian Sarbu, Constantin Mircioiu, Ghaleb Abdalameer Abdalrb, Mirela Adriana Mitu, Emma Adriana Ozon

**Affiliations:** 1 Romanian Academy, IlieMurgulescu Institute of Physical Chemistry, 202 Spl.Independentei, 060021 Bucharest, Romania; iatkinson@icf.ro (I.A.); vtpopa@icf.ro (V.T.P.); 2 Department of Physical and Colloidal Chemistry, Faculty of Pharmacy, “Carol Davila” University of Medicine and Pharmacy, 020956 Bucharest, Romania; valentina.anuta@umfcd.ro; 3 Department of Pharmaceutical Physics and Biophysics, Drug Industry and Pharmaceutical Biotechnologies, Faculty of Pharmacy, “TituMaiorescu” University, 004051 Bucharest, Romania; 4 Department of Applied Mathematics and Biostatistics, Faculty of Pharmacy, “Carol Davila” University of Medicine and Pharmacy, 020956 Bucharest, Romania; constantin.mircioiu@umfcd.ro (C.M.); ghalibalbaag@gmail.com (G.A.A.); 5 Department of Pharmaceutical Technology and Biopharmacy, Faculty of Pharmacy, “Carol Davila” University of Medicine and Pharmacy, 020956 Bucharest, Romania

**Keywords:** captopril, β-cyclodextrin, inclusion complex, physical-chemical characterization, release kinetics

## Abstract

Captopril is the first angiotensin I-converting enzyme inhibitor widely used for the treatment of hypertension. Based on the well-known benefits of cyclodextrin inclusion complexes, the present study investigated the ability of β-cyclodextrin to include captopril. Solid inclusion complexes of captopril with β–cyclodextrin in a 1:2 molar ratio were prepared by using the paste method of complexation. For comparison purposes, a simple physical mixture with the same molar ratio was also prepared. Fourier-transform infrared spectroscopy, scanning electron microscopy, X-ray diffraction and simultaneous thermal analysis were used to characterize the raw materials, physical mixture and solid inclusion complex. In order to provide the drug in a more accessible and patient-compliant form following masking its bitter taste, as well as ensuring the appropriate release kinetics, the investigated complex was formulated as orally disintegrating tablets. The study of captopril dissolution in both compendial and simulated saliva media suggested the Noyes Whitney model as the best mathematical model to describe the release phenomena. A clinical study on healthy volunteers also highlighted the taste improvement of the new formulation as compared to conventional tablets.

## 1. Introduction

Captopril (CAP), (2S)-1-[(2S)-2-methyl-3-sulfanylpropanoyl)] pyrrolidine-2-carboxylic acid, is an oral antihypertensive drug with an elimination half-life following a single oral dose ranging from approximately 1.7 to 2 h [1].

The drug is stable at pH = 1.2, and, as the pH increases, it becomes unstable and undergoes a degradation reaction [2,3], mainly oxidative degradation of the sulfhydryl group [4,5]. In vivo, CAP is extensively converted to metabolites, of which the majority are the mixed disulfide conjugates with either low molecular weight endogenous thiols (cysteine and glutathione) or with proteins [6,7].

CAP is included in class III of the Biopharmaceutical Classification System (BCS), and therefore, it has low membrane permeability and, subsequently, a decreased absorption, whereas dissolution occurs very rapidly [8].

Difficulty in swallowing (dysphagia) is a frequent disorder, especially in elderly, children and psychiatric patients [9]. In such cases, a formulation as orally disintegrating tablets may overcome swallowing limitations and improve patient compliance. Additionally, a rapid disaggregation guarantees a very rapid onset of the effect [10]. Therefore, several CAP orally disintegrating tablets formulations (ODTs) using superdisintegrants were proposed [11,12]. However, the rapid onset of the pharmacological effect associated with sublingual absorption is associated with rapid drug elimination and, therefore, only a short duration of action. Formulations using a mixture of superdisintegrants proved to assure a better bioavailability than standard CAP tablets and pharmacological effects for about 4 h after administration [13].

Additionally, in pediatric therapy, drugs are frequently administered as extemporaneous liquid formulations. However, solutions are far less stable than tablets, and hence, a fast-disintegrating formulation proved to be a much more stable and convenient oral solid extemporaneous preparation of CAP as compared to the classical liquid formulations [14].

Based on the well-known benefits of cyclodextrins (CDs) as excipients in tablet formulations [15], CAP–CD inclusion complexes have been investigated by different research groups for years [16,17,18].

In this context, the main objective of the present paper was to investigate the development and characterization of a new CAP orally disintegrating tablet formulation. In order to provide the drug in a more accessible and patient-compliant form following masking its bitter taste, as well as ensuring the appropriate release kinetics, CAP was formulated as an inclusion complex with β-cyclodextrin.

This paper presents the preparation and physicochemical characterizations of the inclusion complex of CAP and β-cyclodextrin (β-CD), the formulation of the ODTs containing the CAP-β-CD complex andthe study and modeling of the release kinetics of the active substances from the formulations, as well as testing the taste improvement of the new formulation as compared to conventional CAP tablets.

## 2. Materials and Methods

### 2.1. Materials

Captopril was purchased from Baoji Guokang Bio-Technology Co., Ltd., (Baoji, China). β-cyclodextrin was purchased from Global Holding Group Co., Ltd. (Ningbo, China). The ethanol and distilled water were of analytical grade. CompactCel^®®^ TC was provided by BIOGRUND GmbH (Huenstetten, Germany). F-MELT^®®^was purchased from Fuji Chemical Industries Co., Ltd. (Toyama, Japan). The magnesium stearate used in the study was produced by Chris Co Chemical (Lawrence, KS, USA).

### 2.2. Synthesis of the Inclusion Complex

CAP (0.125 mmol) and 0.25 mmol of β-CD, in a 1:2 molar ratio, were mixed together thoroughly in a mortar with vigorous trituration for about 3 h. During this process, a volume of 70% ethanol solution was added until a homogeneous paste was obtained. The resulted paste was further triturated for one hour. Then, the obtained product was dried at room temperature for 24 h. In order to obtain a fine and uniform powder, the solid paste was passed through suitable sieves.

### 2.3. Physical–Chemical Characterization

CAP and β-CD, the processed β-CD, the physical mixture and the inclusion complex, were evaluated by Fourier-transform infrared spectroscopy (FTIR, (Jasco International Co. LTD. Tokyo, Japan) scanning electron microscopy (SEM, Thermo Fisher, Waltham, MA, USA) a simultaneous thermal analysis (STA, Netzsch-Geratebau GmbH, Selb, Germany)-) and X-ray diffraction (XRD, (Rigaku, Tokyo, Japan)). FTIR spectra were recorded using a JASCO FT/IR-4200 spectrometer (Jasco International Co. LTD. Tokyo, Japan) with an ATR PRO450-S accessory (Jasco International Co. LTD. Tokyo, Japan) within a spectral range of 4000–400 cm^−1^ and a resolution of 4 cm^−1^. SEM measurements were carried out on a FEI Quanta 3D FEG microscope (Thermo Fisher, Waltham, MA, USA Powder X-ray diffraction patterns were recorded on a RigakuUltima IV diffractometer ((Rigaku, Tokyo, Japan) with CuKα radiation λ = 1.5406 Å, in the 2θ = 5–60° range, with a scan speed of 5°/min and a 0.02 step size, at 40 kV and 30 mA. Rigaku’s PDXL software (version 1.8), connected to the ICDD-PDF-2 database was used for phase identification. Thermal measurements (TG/DTG-thermogravimetric analysis and differential thermogravimetry; and DSC-differential scanning calorimetry) were performed in dynamic argon atmospheres with a Netzsch STA 449 F1 Jupiter simultaneous thermal analyzer (Netzsch-Geratebau GmbH, Selb, Germany), at a heating rate of 5 °C/min, in the range of 30–600 °C. The accurately weighed samples were placed in uncovered Al_2_O_3_ pans. The samples were analyzed in duplicate.

### 2.4. Development of Pharmaceutical Formulation

Orally disintegrating tablets containing the CAP-β-CD complex equivalent to 25-mg CAP/tablets were prepared by direct compression using a superdisintegrant, F-MELT^®®^, which ensures good flow properties, a dried binder (CompactCel^®®^ TC) and a lubricant (magnesium stearate) in the proportions shown in Table 1.

### 2.5. Study of the Release Kinetics of CAP from the Tablets

Release experiments were performed using the USP basket apparatus (ERWEKA DT800 HH model, Heusenstamm, Germany) at a rotation speed of 50 rpm, as specified in the Captopril Tablet monography of USP32 [19].

Both the official USP dissolution medium (900 mL of 0.01-N HCl) and a more relevant medium simulating the composition of the saliva [20,21] containing 8-g/L NaCl, 0.19-g/L KH_2_PO_4_ and 2.38-g/L Na_2_HPO_4_ with a pH of 6.8 were used.

Samples (2 mL) were collected at 5, 10, 15, 20 and 30 min after the start of the assay. The resulting samples were quantitatively analyzed using a validated HPLC method. All the assays were performed in triplicate.

### 2.6. Taste Evaluation

The prepared tablets were subjected to a taste evaluation in 6 healthy volunteers, who received previous details about the purpose, any risk involved and the procedure for the taste evaluation. The taste evaluation of CAP-β-CD tablets for oral dispersion was conducted in comparison with the CAPOTEN^®®^ 25-mg (Bristol Meyer Squibb, New York, NY, USA) commercial tablets. The protocol of the experiment had the approval no. 155NI/25.09.2019 from the National Bioethical Committee of Medicines and Medical Devices. The volunteers were asked to hold one CAP tablet in their mouths for 20 s, and the sensations felt were recorded. After 20 s, they were asked to spit out the contents and to rinse their mouths using water. After 30 min, they repeated the procedure using CAP-β-CD tablets.

Volunteers had to rank from 1 to 5 the following taste characteristics: bitter taste, grittiness, other taste, residual taste in mouth, acceptance and sulfurous taste. Bitter taste was considered the primary endpoint, and other parameters, secondary endpoints. A numerical scale was used for the scoring of taste, with the following values: 0—tasteless, 1—pleasant, 2—slightly sweet, 3—slightly bitter, 4—moderately bitter and 5—intensely bitter.

### 2.7. Applied Release Theoretical Models

For describing the evolution over time of the cumulative amount of CAP released and to estimate the in vivo release, four mathematical models of release kinetics from solid dosage forms were applied: Higuchi square root law [22], Noyes-Whitney model, the Power-Law (Siepmann Peppas) model [23,24] and the Weibull model [25,26,27].

## 3. Results

### 3.1. Structural, Morphological and Thermal Properties of Compounds

**Figure 1:**
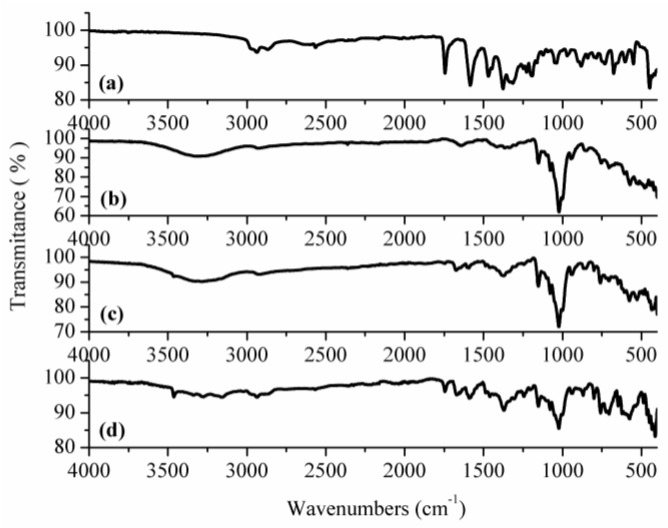
FTIR spectra of the (**a**) captopril (CAP), (**b**) β-cyclodextrin (β-CD) and (**c**) CAP-β-CD physical mixture and (**d**) CAP-β-CD inclusion complex.

FTIR spectroscopy. Figure 1 shows the FTIR spectra recorded for the pure compounds and for the physical mixture and inclusion complex. The fact that no interaction occurred in the physical mixtures between CAP and β-CD was confirmed by FTIR spectroscopy (Figure 1c), which displays only a superposition of the individual bands.

In the FTIR spectrum of CAP, the two bands in the region of 2978 and 2870 cm^−1^ correspond to the –CH_2_ and –CH_3_ groups. A narrow band is observed at 2565 cm^−1^, which is characteristic for the thiol (–SH) group. The C=O vibration of the carboxyl group gives an intense band at 1744 cm^−1^, whereas the corresponding one of the amide group is evidenced by an intense band at 1584 cm^−1^. One may also notice the lower intensity of the characteristic band of the carboxylic O–H group, due to the intermolecular hydrogen bonds (Figure 1a). The results are in good agreement with the literature data [28,29].

The FTIR spectrum of β-CD displays a large band around 3330 cm^−1^, due to the O–H stretching vibrations from the primary or secondary hydroxyl groups, which are connected by the intermolecular and intramolecular hydrogen bonds, respectively [30]. Other strong bands at 1021 cm^−1^ and 1023 cm^−1^ are produced by the C–O bond vibrations (Figure 1b). The FTIR spectrum of the CAP-β-CD inclusion complex (Figure 1c) contains a series of changes in comparison with those of the two single components and of their physical mixture. The spectral range characteristic for captopril significantly decreases in intensity, especially within the 1750–1300cm^−1^ zone, due to the inclusion of CAP in the β-CD cavity. The characteristic O–H band is narrower, increases significantly in intensity and shifts to around 3460 cm^−1^, due to the inclusion of CAP in the CD cavity which forms new intermolecular hydrogen bonds between the two compounds.

**Figure 2:**
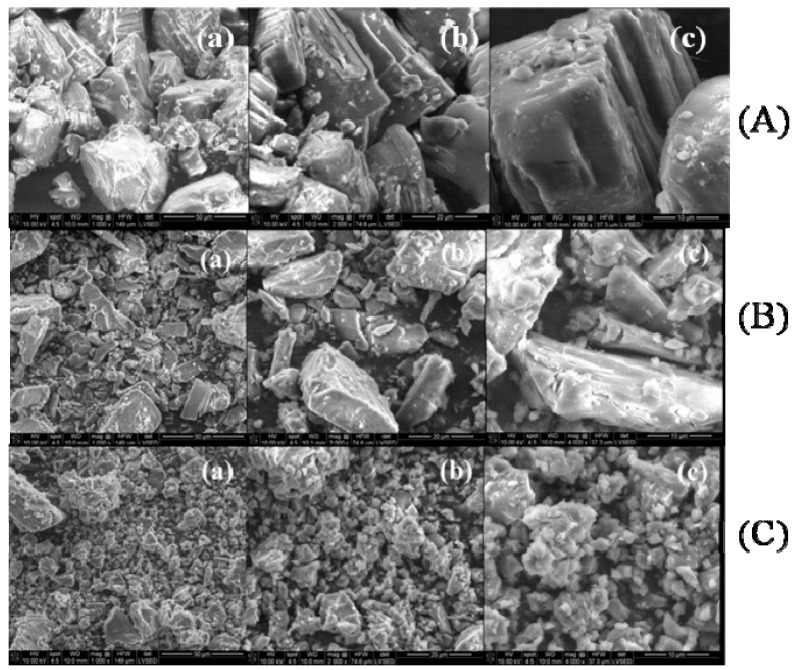
SEM images of (**A**) CAP at different magnifications: (**a**) 1000×, (**b**) 2000× and (**c**) 4000×; (**B**) β-CD at different magnifications: (**a**) 1000×, (**b**) 2000× and (**c**) 4000×and (**C**) processed β-CD at different magnifications: (**a**) 1000×, (**b**) 2000× and (**c**) 4000×.

**Figure 3:**
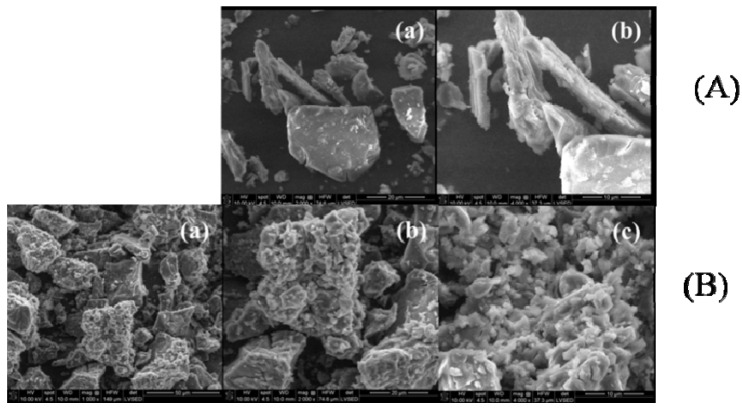
SEM images of (**A**) the CAP-β-CD physical mixture at different magnifications: (**a**) 2000× and (**b**) 4000×, and(**B**) the CAP-β-CD inclusion complex at different magnifications: (**a**) 1000×, (**b**) 2000× and (**c**) 4000×.

SEM analysis. There are several interesting peculiarities of the SEM measurements that should be highlighted. The SEM images at different magnifications (between 1000× and 4000×) of CAP, β-CD, processed β-CD, the physical mixture and the CAP-β-CD inclusion complex are presented in Figure 2 and Figure 3. CAP (Figure 2A) is characterized by the presence of scratchy crystalline particles, with sizes between 3 and 50 μm.

The literature data indicate that β-CD crystallizes in polyhedral forms [16]. SEM images at different magnifications of β-CD showed different shapes of homogenous crystalline particles, with sizes between 5 and 30 μm (Figure 2B). The processed β-CD also displays crystalline particles, more homogenous in shape, with sizes between 3 and 10 μm. The significant reduction in the particle size (Figure 2C) is due to the processing method.

The SEM images of the CAP-β-CD physical mixture (Figure 3A) exhibit the morphological features of both CAP and β-CD but with no evidence of a newly formed compound. SEM micrographs show only both crystalline components.

In contrast, the SEM micrographs of the CAP-β-CD inclusion complex (Figure 3B) signal the formation of a new structure, the complex appearing as very fine and irregular crystals, with an obvious decrease of the particle size and a partial loss of crystallinity in comparison with the initial components. The SEM micrographs clearly suggest the formation of the CAP-β-CD inclusion complex, thus signing for the affinity of CAP versus β-CD. From the SEM analysis, the existence of components and information about the particle shape and size was confirmed, in agreement with the literature data [16,31]. The obtained micrographs of the inclusion complex showed that it was not possible to differentiate the structures of the individual components, indicating a strong interaction between CAP and β-CD [31].

**Figure 4:**
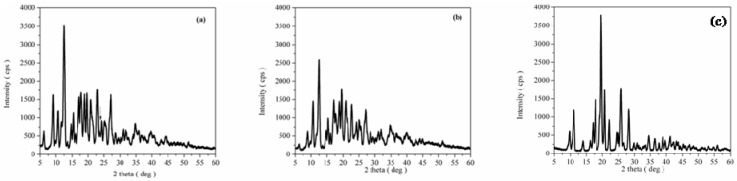
X-ray diffraction (XRD) pattern of (**a**) β-CD, (**b**) processed β-CD and (**c**) CAP.

**Figure 5:**
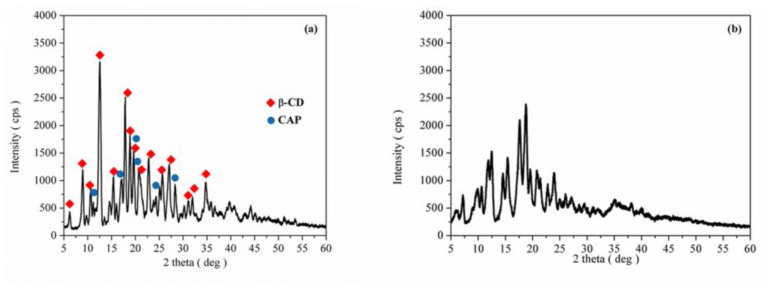
XRD pattern of (**a**) the CAP-β-CD physical mixture and (**b**) the CAP-β-CD inclusion complex.

XRD analysis. Powder X-ray diffraction patterns of CAP, β-CD, processed β-CD, the CAP-β-CD physical mixture and the CAP-β-CD inclusion complex are presented in Figure 4 and Figure 5. Both CAP and β-CD present numerous sharp diffraction peaks, suggestive of their crystalline state.

The X-ray diffraction pattern of β-CD (Figure 4a) confirms its crystalline form, with characteristic peaks at 2θ diffraction values 9.01°, 12.51°, 17.80°, 19.60°, 22.78°, 24.33°, 27.04° and 35.88°. All the diffraction lines could be indexed to beta cyclodextrindecahydrate C_42_H_70_O_35_-C_7_H_7_NO_2_·10H_2_O (JCPDS card 00-054-1476) with a monoclinic structure. The powder X-ray diffraction pattern of processed β-CD (Figure 4b) looks like the one of simple β-CD but reduced in their intensity, indicating the fact that the paste process has no major influence on the compound’s crystalline structure.

The XRD diffraction pattern of pure CAP (Figure 4c) shows its highly crystalline nature, indicated by numerous distinctive diffraction lines according to JCPDS card 00-035-1916, in agreement with the literature data [32]. The 2θ diffraction values of the more intensive peaks are 2θ = 11.11°, 17.86°, 19.60°, 25.89° and 28.27°.

The powder X-ray diffraction pattern of the CAP-β-CD physical mixture (Figure 5a) reveals the presence of β-CD as a major phase and captopril. The formation of a new compound with a new structure was not emphasized by XRD analysis. The XRD diffraction pattern of the physical mixture from Figure 5a was found to be the simple superposition of the XRD of each component. Figure 5b presents the powder X-ray diffraction pattern of the CAP-β-CD inclusion complex, showing a significant decrease in the degree of crystallinity, as evident from the disappearance of sharp distinctive peaks keeping somehow the base characteristics of the initial β-CD. There are several peaks that are not present in the diffractogram of pure β-CD, suggesting that a new phase was being formed. These observations may be attributed to an interaction between CAP and β-CD, suggesting the presence of a new solid phase with lower crystallinity than the initial components. The XRD evidence of this new crystalline phase indicates a most plausible binding of CAP inside the CD cavity.

**Figure 6:**
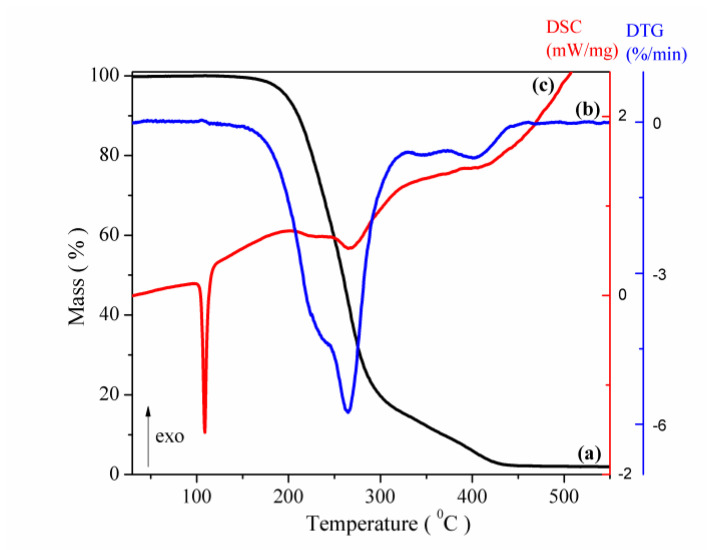
Thermal curves of the CAP (**a**) TG, (**b**) DTG and (**c**) DSC (dynamic argon atmosphere, β = 5 °C min^−1^).

Thermal analysis. The thermal curves (TG/DTGand DSC) of CAP are shown in Figure 6. The DSC curves of CAP exhibits a sharp endothermic peak at 103 °C (peak temperature of DSC curve recorded at 5 °C min^−1^), corresponding to its melting point. This value is in very good agreement with the published ones [16,25,30,32,33]. The shape of the peak is an indication of its purity and crystalline state. The melting enthalpy evaluated from the peak area is Δ*H* = 102.7 J g^−1^ (22.3 kJ mol^−1^), comparable with those reported in the literature for captopril [28,33,34,35,36,37].

During heating in the argon atmosphere, after the melting process (indicated by the DSC curve, Figure 6c, the TG curve of CAP shows a mass loss of 97.9% in the temperature range 156–450 °C and a small residue of carbon (2.2%) was left at the end of the experiment. As expected, the decomposition is associated with a broad endothermic effect (on the DSC curve). The onset weight loss temperature calculated from the TG curve (Figure 6a) was around 156 °C, followed by the second weight-loss step between 310 and 450 °C. The first mass loss of 82.5% (between 156 and 310 °C) was assigned to the methyl and carboxylic groups. The second mass loss of 15.2% (between 310 and 450 °C) was attributed to the SH loss and pyrolysis of the molecule [29].

**Figure 7:**
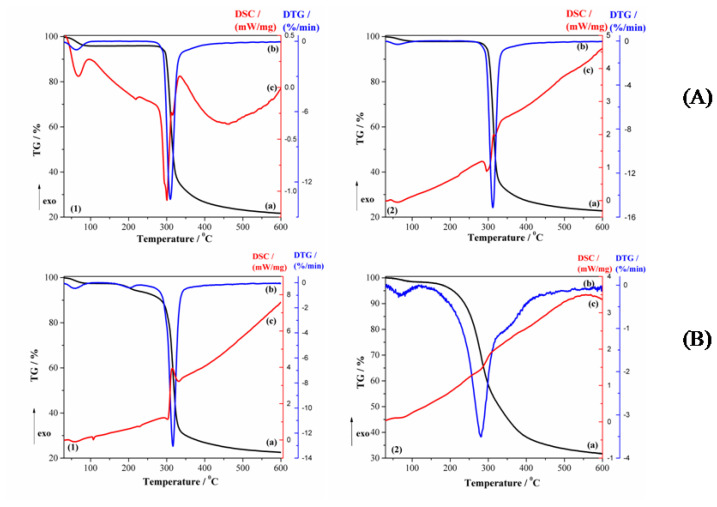
Thermal curves (TG, DTG and DSC) in (**A1**) β-CD (**a**) TG, (**b**) DTG and (**c**) DSC and (**A2**) processed β-CD (a) TG, (b) DTG and (c) DSC (dynamic argon atmosphere, β = 5 °C min^−1^) and (**B1**) the CAP-β-CD physical mixture (a) TG, (b) DTG and (c) DSC and (**B2**) the CAP-β-CD inclusion complex (a) TG, (b) DTG and (c) DSC (dynamic argon atmosphere, β = 5 °C min^−1^).

Both the DSC curves of β-CD and of processed β-CD (Figure 7A) show an endothermal peak at 69.4 °C for β-CD and 64.1 °C for processed β-CD, respectively, followed by a small endothermal peak, without any weight loss (*T*_DSC_ = 214 °C for β-CD and 222 °C for processed β-CD), which can be attributed to a reversible structural solid-solid phase transformation [37,38]. The first endothermic peak with a low intensity, which appeared below 90 °C, is due to the absorbed water molecules from the outside and inside of the β-CD cavity [39]. The mass loss in the temperature range 30–90 °C (TG curves from Figure 7A are about 4.1% for β-CD (*T*_DTG_ = 60.6 °C) and 1.9% for processed β-CD (*T*_DTG_ = 63.2 °C). The melting process of β-CD occurs in the temperature range 250–301 °C (*T*_DSC_ = 300.9 °C), the endothermal effect from the DSC curve (Figure 7A), according with the literature data [38], but the melting and decomposition processes are superposed. The melting peak of processed β-CD appears at a lower temperature (*T*_DSC_ = 295 °C) and with much lower intensity. Finally, the degradation process took place in the temperature range 270–340 °C (*T*_DTG_ = 309.7 °C for β-CD and *T*_DSC_ = 318 °C and *T*_DTG_ = 312 °C for processed β-CD) (Figure 7A). The degradation process shows a mass loss of 62.5% for β-CD and 65.1% for processed β-CD. At higher temperatures, a continuous pyrolysis occurs from 340 to about 600 °C. The final residue is 21.7% for β-CD and 22.8% for processed β-CD.

The thermal curves (TG/DTG and DSC) of the CAP-β-CD physical mixture (Figure 7(B1)) showed the superposition of the thermal behavior of individual CAP and β-CD molecules. The characteristic melting peak of CAP was shifted from 103 °C to 107.4 °C and its intensity decreased. The melting peak of β-CD remained at the same value, but its intensity was reduced. The peak corresponding to the structural transformation solid-solid phase transition of β-CD was shifted from 214 °C to 228.2 °C. From the TG curves, the mass loss between 30 and 90 °C is 2.5% (*T*_DSC_ = 58.4 °C and *T*_DTG_ = 55.6 °C), which is attributed to water molecules. The degradation process took place between 160 and 600 °C (*T*_DSC_ = 316.3 °C and *T*_DTG_ = 315.4 °C), with a total mass loss of 74.9%. The residue is about 22.6%. The above results show the absence of an interaction between captopril and β-CD, which proves that the complexation phenomenon did not occur, in complete accordance with the results from the XRD analysis.

**Figure 8:**
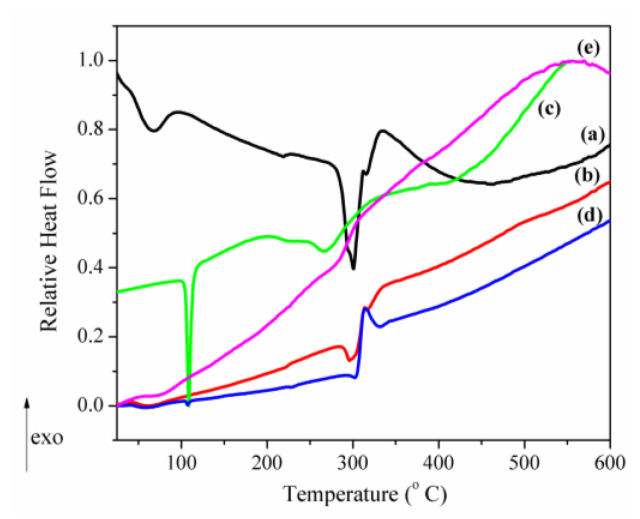
The relative heat flow DSC curves of (**a**) β-CD, (**b**) processed β-CD, (**c**) CAP, (**d**) the CAP-β-CD physical mixture and (**e**) the CAP-β-CD inclusion complex.

It is well-known that, when a guest molecule is encapsulated in CD cavities, its physical properties such as melting, boiling or sublimation points are generally shifted to different temperatures than the parent compound(s) or disappear [16,40,41]. Therefore, the disappearance of the CAP and β-CD melting points in the CAP-β-CD complex suggests the formation of an inclusion complex. The TG curve from Figure 7(B2) shows that the dehydration process of the inclusion complex is completed at a much higher temperature (76.2 °C) and with a mass loss (1.7%) lower than the parent β–CD. This is a confirmation that, during the inclusion process, most of the water molecules from the β-CD cavity are replaced by the CAP molecules. The DSC curve of the CAP-β-CD inclusion complex (Figure 7(B2)) showed only one large exothermic peak at 306.9°C and the complete disappearance of the structural transformation of β-CD. The DSC thermal features of the two components are practically erased in the thermogram of the inclusion complex. This is clearly signing for a new compound. For comparison purpose, a “relative heat flow”, RHF, was calculated as described in Equation (1):

(1) $$\mathrm{RHF}=\frac{\mathrm{HF}\left(T\right)-{\mathrm{HF}}_{\min}}{{\mathrm{HF}}_{\max}-{\mathrm{HF}}_{\min}},$$

where RHF is the relative heat flow, *T* is the temperature of the thermal process, HF(*T*) is the observed heat flow (the DSC output), HF_min_ is the minimum heat flow signal and HF_max_ is the maximum heat flow signal. This brings all the experimental signals within the range 0 ≤ HRF ≤ 1. The data are represented in Figure 8.

Figure 8 contains several apparent features:

(i) The marked attenuation (in the case of the physical mixture) or absence (in the case of the inclusion complex) of a CAP-melting endotherm. This is a sign that a consistent amount of CAP is included into the β-CD cavity, even within the 15-min mortar mixing procedure, the process being complete for the inclusion complex. The above assumption is also supported by the lower mass loss associated with “cavity-included” water in the case of the physical mixture, compared to the pure cyclodextrin.
(ii) The decrease of the β-CD phase transition endotherm for both processed β-CD and its physical mixture with CAP and the absence of this endotherm for the inclusion complex.
(iii) The decrease of the endo-exothermogram feature in the order β-CD > processed β-CD > physical mixture > inclusion complex. This is the thermal signature associated with the melting-decomposition of cyclodextrine; in part, the latter process takes place on behalf of the provided intramolecular oxygen. In the case of the physical mixture, this cyclodextrine oxygen may act on the CAP component. In the case of the inclusion complex, this endo-exo feature is severely attenuated.

All of the above facts point to a gradual loss crystalline structure identity of both CAP and β-CD, due to either physical mixing or paste method processing. This process, supported by XRD data, is “complete” in the inclusion complex, whose new chemical and crystalline identity is proved by FTIR, XRD and STA. This assumption is also supported by the total mass loss of the inclusion complex, which was slightly lower than that of the physical mixture (Δm = 9.1% at 600 °C). This is due to the fact that the independent β-CD is still present in the physical mixture of CAP-β-CD, on one hand. On the other hand, because of the higher thermal stability of the inclusion complex, the exothermic decomposition on behalf of the active oxygen species “donated” by the *β*-CD is hindered in the complex, as compared to the physical mixture.

### 3.2. Release Kinetics

Several authors have sought to achieve the rapid disintegration and release of CAP from tablets. Karaya gum in the concentration of 9% *w*/*w* with Avicel PH 102 in 25% *w*/*w* gave rapid disintegration in 25 sec and showed a 100% drug release within 5 min [12]. Besides the utilization of superdisintegrants, CAP was replaced with the CAP-β-CD complex in order to reduce the bitter taste of CAP. A solid dispersion of the drug with β-cyclodextrincrospovidone, croscarmellosesodium and sodium starch glycolate was formulated. The superdisintegrantcrospovidone in the concentration of 10% gave the fastest disintegration in 20 s and showed 92% drug release in 5 min. Volunteers’ opinions concerning the taste was rated by giving different score values. In the taste evaluation, 100% of volunteers reported that the solid dispersion of captopril: β-cyclodextrinat a 1:3 ratio was tasteless, but 70% of volunteers reported that it was palatable at a 1:2 ratio [42].

Inclusion complexes of CAP-*β*-cyclodextrin, by using different polymers with superdisintegrants like microcrystalline cellulose and croscarmellosesodium, lead to tablets with in-vitro disintegration times in the range of 15 to 48 s. The authors claimed a diminishingin the bitter test, but this effect was not tested [43].

The CAP-β-cyclodextrin complex with different polymers was formulated to mask the bitter taste and to obtain rapid dissolution. A drug release of 99.86 ± 0.54% was obtained in 12 min, but an improvement of the taste was not verified [44].

#### 3.2.1. Release in Compendial Medium

**Figure 9:**
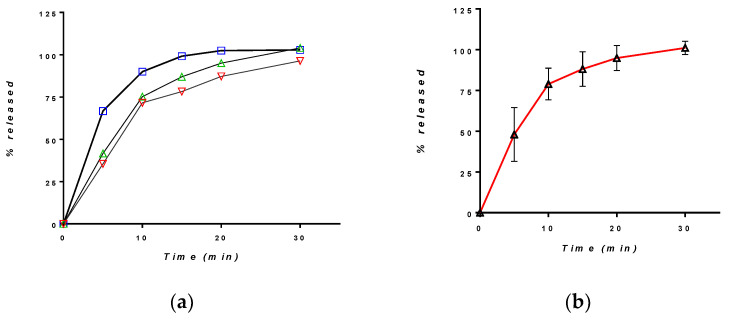
Release of CAP from the CAP-β-CD in HCl 0.01N (**a**) individual curves and (**b**) mean profile.

The dissolution of CAP-β-CD in HCl 0.01N was evaluated using the compendial USP method for CAP tablets. The individual and mean release curves are depicted in Figure 9.

**Figure 10:**
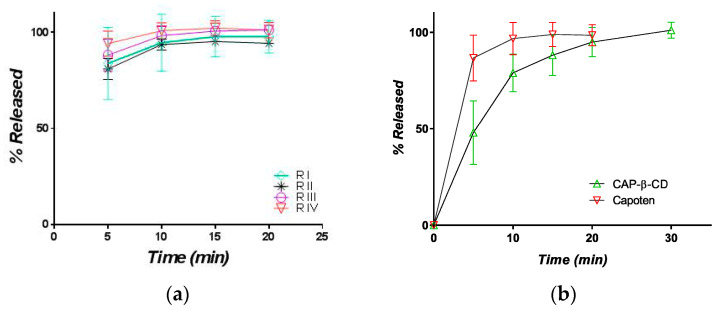
Mean release profile of CAP in HCl 0.01N from (**a**) 4 different CAPOTEN^®®^batches and (**b**) a comparative profile between CAPOTEN^®®^ and the experimental orally disintegrating tablets containing CAP-β-CD.

The dissolution was rapid, with a moderate variability between the individual profiles, but complete after 30 min. For comparison, four different batches of the brand captopril, CAPOTEN^®®^ 25 mg Bristol Meyers Squibb (Figure 10), were tested.

The inter-batch variability was low, and the release was more rapid. A comparison between the mean dissolution curves of the experimental tablets and CAPOTEN^®®^ shows that dissolution from CAP-β-CD was slower and lasted some ten minutes more. However, following the official criteria for the comparison of dissolution curves [41], more than 85% of the active substance was released within the first 15 min, and the formulations have a similar dissolution profile in 0.01N.

#### 3.2.2. Release in Simulated Saliva

**Figure 11:**
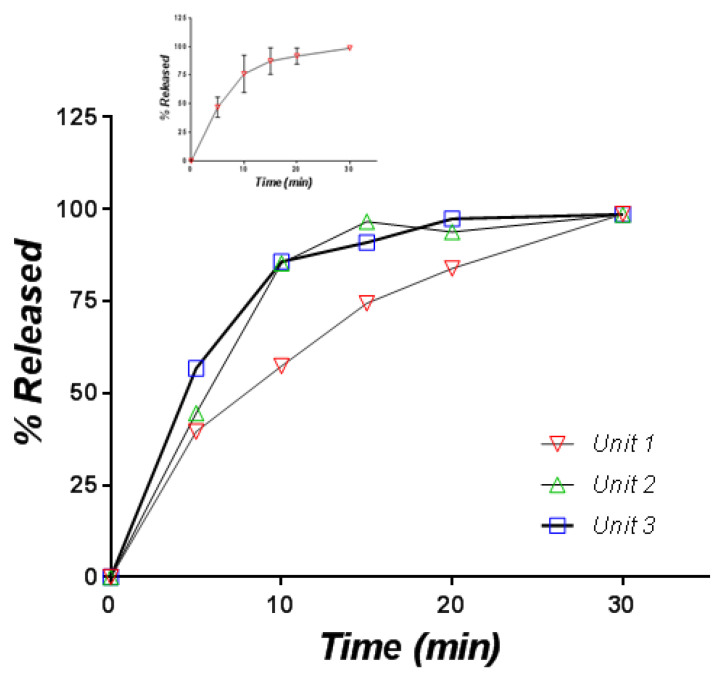
Individual and mean (inset) in vitro release profiles of captopril from orally disintegrating tablets containing the CAP-β-CD inclusion complex (medium: simulated saliva, 900 mL).

Since the formulation was conceived as orally disintegrating, the release of CAP in a simulated saliva medium is theoretically more biorelevant than the release in an acidic medium. The release curves are presented in Figure 11.

**Figure 12:**
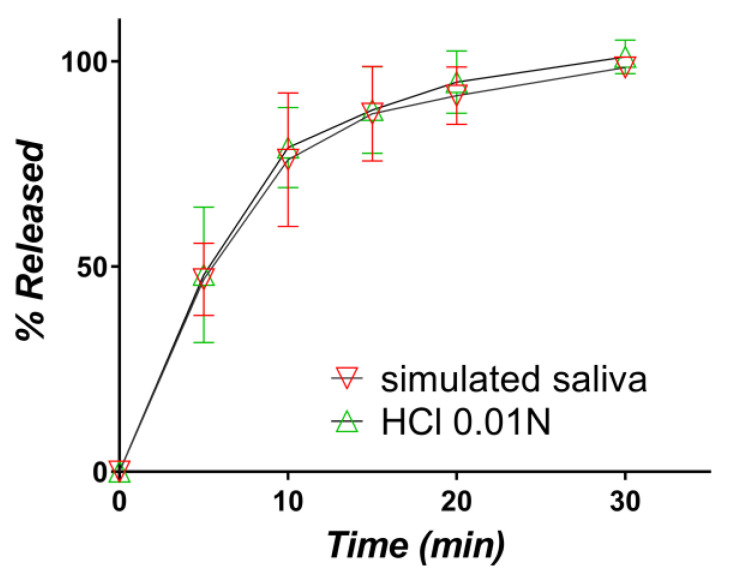
Comparative assessment of the in vitro release profiles of captopril from the orally disintegrating tablets containing the CAP-β-CD inclusion complex in simulated saliva (red) and 0.01 N (green) HCl, respectively.

Variability is again present, one curve being significantly under the others, but at 30 min, the release was complete in all three cases. Finally, the release in the simulated saliva and HCl 0.01 N were practically identical, as can be seen from a comparison of the mean curves (Figure 12).

### 3.3. Selection of the Most Appropriate Mathematical Model

In order to estimate the mechanism of release of the active substance, the profiles obtained were evaluated using the mathematical analysis connected with the mentioned phenomenological or empirical kinetic models.

The assessment of the captopril release mechanism from tablets containing CAP-β-CD inclusion complexes by mathematical modeling was performed using the DDSolver Excel add-in software package [45].

The performance of the mathematical models in describing the experimental data was evaluated by comparing the mean of squared errors and, also, by applying the Akaike Information Criterion (AIC). The model with the lowest AIC was considered the best estimation of the time evolution of the release [45,46].

In modeling, it was taken into consideration that the release could start later than the moment zero, appearing as a so-called time-lag (Tlag).

**Figure 13:**
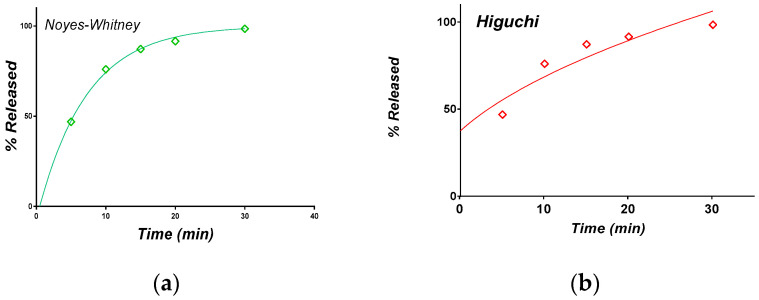

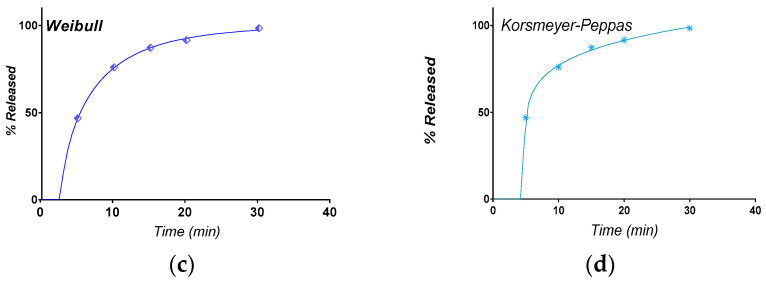
Fitting of the CAP release experimental profiles from orally disintegrating tablets containing the CAP-β-CD inclusion complex in simulated saliva, using various mathematical models: (**a**) Noyes-Whitney, (**b**) Higuchi, (**c**) Weibull and (**d**) Korsmeyer-Peppas.

The fitting of the captopril release profiles from the orally disintegrating tablets containing the CAP-β-CD inclusion complex into the simulated saliva using the above models is shown in Figure 13.

The parameters of each model, as well as the performance evaluation of the fittings, are shown in Table 2.

Following the software approach, the best model is Weibull, followed by Korsmeyer-Peppas. It is to make the observation that, in terms of mathematical expression, the model Korsmeyer-Peppas is the same with the SiepmannPeppas model and with the Peppas model. Differences concern the phenomenological conditions in the deriving models, but it is clear that the software uses only the mathematical “power-law model”, being blind to these conditions.

**Figure 14:**
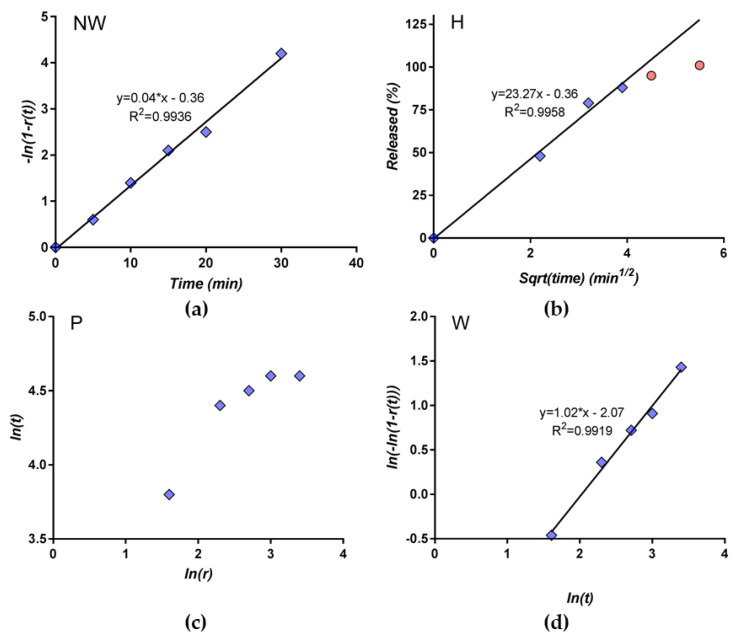
The linear regression of the transformed data: (**a**) the Noyes-Whitney model, (**b**) Higuchi model, (**c**) Peppas model and (**d**) Weibull model.

An alternative approach to modeling and to evaluating the performances was based on examining the transformed data in order to express dependencies in a linearized form. The models were used in linearized forms, as was previously described [47] (Figure 14): (i) ln(1-r(*t*) as a function of t for the Noyes-Whitney model(NW), (ii) ln(-ln(1-r(*t*)) as a function of ln(*t*) for theWeibull model (W) and (iii) ln r(*t*) as a function of ln(*t*) for the Peppas model (P). The good linear correlation in the case of the NW and W models is noted. The Higuchi model works for an 80% release, but this is maximum value is expected in the case of this model. The attempt to fit the entire set of data made by software is an abnormal approach. The first point is not well-estimated by the power-law Peppas model.

### 3.4. Comparison of Release Kinetics Models

The software modeling procedure was, in fact, similar to the usual analysis of the release kinetics from the supramolecular systems: the trying of more models and establishing a hierarchy, mainly as a function of the correlation coefficient [47] or, sometimes, of the AIC. Such an approach was also applied by other authors in modeling the release from captopril-retarded formulations [48].

The alternative method, based on the linearized forms of models, considers the Noyes-Whitney model as the most performant. Since in the Peppas model dissolution and diffusion are considered inside the tablet matrix, and Noyes-Whitney considers diffusion outside the matrix through the interface, the results of the two analysis methods are contradictory. These differences between phenomenological conditions led to different initial and boundary conditions for the associated equations and to different solutions. As presented in an extensive review of one of the authors [47], these solutions are usually verified in the literature as mathematical models, without checking the phenomenological conditions.

It is to note that, for improving the fitting of the data with the Peppas equation, the software method introduced a time-lag, i.e., introduced a new parameter corresponding to an event with very low probability.

The Noyes-Whitney model is a particular “order 1” kinetics model, better-defined and more mechanistic. Examining Table 2, the value of the AIC criterion in the case of order 1 kinetics is not too far from that in the case of the Peppas model. Once again, the Noyes-Whitney model appears as the most probable one. The software empiric analysis, “blind” towards the phenomenological conditions, remains a usual, but less significant, analysis.

The dissolution methods and models applied to the analysis of the results are looked to be “biorelevant”, i.e., to be closer to in vivo conditions and to allow an estimation of the in vivo dissolution and pharmacokinetics [49,50]. For this reason, it was applied, additionally to the compendial method, to the release in the simulated saliva. Since the release curves were practically identical, the compendial method could be considered sufficiently biorelevant.

### 3.5. Evaluation of Taste

As can be seen in Table 3, from six healthy volunteers who evaluated the taste of the tablets:(i) three of them judged CAP-β-CD tablets for oral dispersion as grade 1 (pleasant), (ii) two of them as grade 2 (slightly sweet) and (iii) one as grade 3 (slightly bitter). All of them assessed the orodispersable tablets as having a slightly pleasant taste, no roughness being reported. On the contrary, the CAP tablets had a burning, metallic taste.

The conclusion is that the inclusion of captopril in cyclodextrins significantly improves all five components of the taste of the tablets.

## 4. Conclusions

A pharmaceutical formulation of CAP and β-CD in a molar ratio of 1:2 was accomplished by the paste method of complexation in a solid state. The same physical-chemical investigations were performed on: nonprocessed CAP, nonprocessed β-CD, processed β-CD, a physical mixture between CAP and β-CD with no processing and the inclusion complex. FTIR spectra revealed that the inclusion complex has strong bonds, proved by significant increases in the intensity of the bound O–H band. The morphology of the investigated specimens, evaluated by SEM, revealed a remarkable change of the shape, crystallites sizes and structure of the new pharmaceutical formulation compared to the single components and their physical mixture. The powder X-ray diffraction patterns proved that the β-CD fingerprint is still present within the physical mixture, while that of CAP is considerably weakened. On the other hand, the XRD pattern of the novel compound obtained by the paste complexation process revealed the formation of a new compound with some of the β-CD characteristics. The thermograms registered in the temperature range between 30 and 600 °C showed that the complexation was complete, as evidenced by the total disappearance of the CAP and β-CD melting peaks. A corroboration of all the analytical techniques strongly supports that the CAP inclusion complex with *β*-CD in a 1:2 molar ratio was successfully prepared by the paste method. The results of the present study prove that the CAP-β-CD inclusion complex can be embedded as an active ingredient of sublingual tablets that can be subsequently used in therapeutic practice.

CompactCel^®®^ TC as dry binder and F-MELT^®®^ as super-agglomerating, directly compressible excipients, both with good flow properties, allow the obtaining of tablets containing the CAP-β-CD complex.

The release profiles of CAP from tablets based on CAP-β-CD and from the brand CAP drug CAPOTEN^®®^, were similar, the released amount being greater than 85% in 1ess than 15 min.

The modeling of the release kinetics based on the least squares methods, as well as the evaluation of fitting by linearized equations, indicated the Weibull model as the most appropriate, but the model is an empirical one. A phenomenological analysis, combined with statistic and informatics criteria, indicate as a probable mechanism the release of CAP through the interface between the tablet matrix and solvent by diffusion (the Noyes-Whitney model).

CompactCel^®®^ TC as taste-masking agent, F-MELT^®®^is sweetening and flavoring, and the inclusion of CAP in the CAP-β-CD complex modified significantly the taste of the tablets, these appearing to be much more acceptable by patients.

## Figures and Tables

**Table 1:** Composition of the developed captopril-β-cyclodextrin (CAP-β-CD) tablets.

Ingredients	Quantity (mg/tb)	Function
CAP-β-CD (1:2) complex	286.0	active ingredient
F-MELT^®®^	184.0	filler, superdisintegrant
CompactCel^®®^ TC	25.0	dry binder
Magnesium stearate	5.00	lubricant
Total	500.0	

**Table 2:** Evaluation of the performance of the captopril release profiles in simulated saliva (Pk-Solver). AIC: Akaike Information Criterion.

Parameter	Order I	Higuchi	Korsmeyer-Peppas	Weibull
K	k_1_ = 0.143	k_H_ = 18.154	k_KP_ = 59.120	a = 2.934
T_lag_	0.487	‒4.285	4.765	T_i_ = 2.614
			*n* = 0.160	β = 0.713
N_obs	5	5	5	5
Degrees of Freedom	3	3	2	2
Observed correlation coefficient R	0.997	0.922	0.998	0.999
R^2^	0.994	0.849	0.997	0.998
R^2^ adjusted	0.993	0.798	0.995	0.996
Mean Square Error	2.83	82.45	1.73	1.32
Sum of squared errors	8.49	247.36	3.46	2.64
AIC	14.69	31.55	12.21	10.86

**Table 3:** Evaluation by subjects of the taste for the tablets containing CAP and CAP-β-CD.

	Bitter Taste	Grittiness	Residual Taste	Other Taste	Acceptance	Sulfurous Taste
CAP	4	3	4	Burn metallic	1	4
CAP-β-CD	1	1	2	Sweet	4	1
CAP	3	1	4	Burn	1	3
CAP-β-CD	1	3	2	Sweet	3	1
CAP	4	2	4	Burn metallic	1	3
CAP-β-CD	1	2	2	Sweet	3	1
CAP	3	3	4	burn	2	2
CAP-β-CD	1	2	2	sweet	3	1
CAP	2	3	3	metallic	3	1
CAP-β-CD	1	3	2	metallic	4	1
CAP	3	3	5	metallic	2	1
CAP-β-CD	1	2	2	sweet	5	1
CAP	5	3	4	pricking	1	2
CAP-β -CD	1	1	1	-	4	1
CAP	4	4	4	astringent	2	1
CAP-β-CD	1	2	2	-	3	1
CAP	4	3	4	astringent	2	2
CAP-β -CD	1	2	2	-	4	1
CAP	3	2	3	metallic	2	1
CAP-β-CD	1	2	3	-	4	1
CAP	5	3	4	burn	1	3
CAP-β-CD	1	2	2	metallic	4	1
MEAN CAP	3.64	2.73	3.91	-	1.64	2.09
MEAN CAP-β-CD	1.00	2.00	2.00	-	3.73	1.00
MEDIAN CAP	4	2	4	-	3	2
MEDIAN CAP-β-CD	1	4	2	-	2	1
Paired *t*-test, *p*<	0.000001	0.035	0.000009	-	0.000004	0.003
